# (*Z*)-*N*-{(*E*)-10-[(2,6-Diisopropyl­phen­yl)­imino]-9,10-dihydro­phenanthren-9-yl­idene}-2,6-dimethyl­aniline

**DOI:** 10.1107/S1600536812003790

**Published:** 2012-02-04

**Authors:** Dongni Li, Hongmei Yu, Tianhua Yu, Haiying Liang, Tiemei Liu

**Affiliations:** a Department of Blood Transfusion, China–Japan Union Hospital, Jilin University, Changchun 130033, People’s Republic of China

## Abstract

The title compound, C_34_H_34_N_2_, adopts a *Z*,*E* configuration with respect to the N=C—C=N backbone, with an N—C—C—N torsion angle of 41.1 (4)° The dihedral angle between the benzene rings in the 9,10-dihydro­phenanthrene moiety is 18.0 (1)°.

## Related literature

For the synthesis and applications of related α-diimines in catalysis and coordination chemistry, see: Li, Gomes *et al.* (2009 ▶); Li, Jeon *et al.* (2009 ▶); Gao *et al.* (2011 ▶); Bochkarev *et al.* (2010 ▶); Belzen *et al.* (1996 ▶). For standard bond distances, see: Allen *et al.* (1987 ▶).
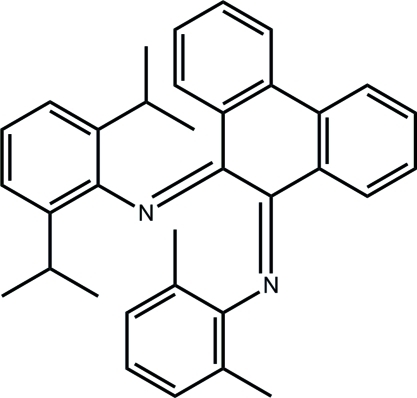


## Experimental

### 

#### Crystal data

C_34_H_34_N_2_
*M* *_r_* = 470.63
Monoclinic, 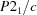

*a* = 9.5495 (7) Å
*b* = 16.4294 (12) Å
*c* = 17.7237 (13) Å
β = 104.579 (1)°
*V* = 2691.2 (3) Å^3^
*Z* = 4
Mo *K*α radiation
μ = 0.07 mm^−1^
*T* = 185 K
0.23 × 0.20 × 0.15 mm

#### Data collection

Bruker SMART CCD area-detector diffractometer
Absorption correction: multi-scan (*ABSCOR*; Higashi, 1995 ▶) *T*_min_ = 0.985, *T*_max_ = 0.990
14490 measured reflections
5304 independent reflections
4587 reflections with *I* > 2σ(*I*)
*R*_int_ = 0.038

#### Refinement

*R*[*F* ^2^ > 2σ(*F* ^2^)] = 0.083
*wR*(*F* ^2^) = 0.161
*S* = 1.27
5304 reflections
331 parameters
H-atom parameters constrained
Δρ_max_ = 0.21 e Å^−3^
Δρ_min_ = −0.24 e Å^−3^

### 

Data collection: *SMART* (Bruker, 1998 ▶); cell refinement: *SAINT* (Bruker, 1998 ▶); data reduction: *SAINT*; program(s) used to solve structure: *SHELXS97* (Sheldrick, 2008 ▶); program(s) used to refine structure: *SHELXL97* (Sheldrick, 2008 ▶); molecular graphics: *SHELXTL* (Sheldrick, 2008 ▶); software used to prepare material for publication: *SHELXTL*.

## Supplementary Material

Crystal structure: contains datablock(s) global, I. DOI: 10.1107/S1600536812003790/lr2046sup1.cif

Structure factors: contains datablock(s) I. DOI: 10.1107/S1600536812003790/lr2046Isup2.hkl

Supplementary material file. DOI: 10.1107/S1600536812003790/lr2046Isup3.cml

Additional supplementary materials:  crystallographic information; 3D view; checkCIF report

## Figures and Tables

**Fig. 1.**
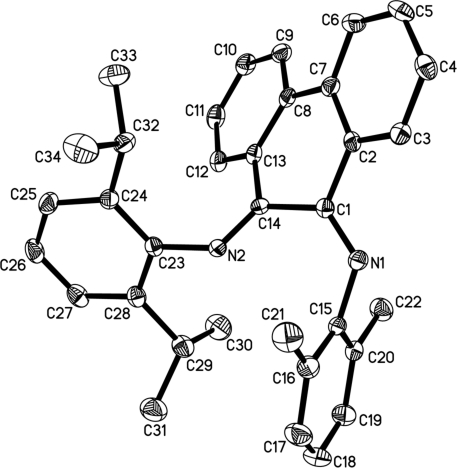
A view of the molecule of the title compound.. Displacement ellipsoids are drawn at the 30% probability level.

## supplementary crystallographic information

### Comment

α-diimines and their metal complexes have been attracted considerable interest due to their applications in catalysis and coordination chemistry. (Li, Gomes *et al.*, 2009; Li, Jeon *et al.*, 2009; Gao *et al.*, 2011; Bochkarev *et al.*, 2010; Belzen *et al.*, 1996). In recent years, we have been interested in the development of high-performance catalyst systems based on diimine ligands and therefore synthesized a series of bis-(arylimino)acenaphthene ligands. Herein, we report the preparation and crystal structure of new phenanthrenequinone-based diimine compound, (I).

The title molecule, Fig. 1, is present as the *Z*, *E* configurational isomer. The C1—C14 distance is 1.507 (3) Å, indicative of no conjugation between the two imine bonds or between the phenyl groups of the phenanthrene backbone. The dihedral angle between the benzene rings of the phenanthrene moiet is 18.0 (1)°. The torsion angle of N1—C1—C14—N2 is 41.1 (4)°. Both lengths and angles in the title compound are in normal ranges (Allen *et al.*, 1987) and are comparable to those of the known phenanthrenequinone-based diimine compounds (Belzen *et al.*, 1996).

### Experimental

To a solution of 2,6-diisopropylaniline (1.08 g, 7.2 mmol) and Dabco (2.49 g, 21.6 mmol) in toluene (30 ml) was added dropwise 7.2 ml of the 1.0 *M* solution of TiCl_4_ in toluene over 30 min at 363 k, followed by addition of a suspension of (*E*)-10-(2,6-dimethylphenylimino)phenanthren-9(10*H*)-one (Gao *et al.*, 2011) (1.5 g, 4.80 mmol) in 10 ml of toluene. The reaction mixture was heated to 413 k for 8 h. The precipitate was removed by hot filtration. The filtrate was evaporated *in vacuo*. The deep red crystalline solid was isolated by silica gel column chromatography (hexane/ethyl acetate, 8:1). (1.08 g, yield: 48%) ^1^H NMR (300 MHz, CDCl_3_, 298 K) δ (p.p.m.): 0.60 (d, *J*_H—H_= 9.0 Hz, 3.6H, CH(CH_3_)_2_), 0.80 (d, *J*_H—H_= 6.0 Hz, 3.6H, CH(CH_3_)_2_), 1.01 (d, *J*_H—H_ = 6.0 Hz, 2.4H, CH(CH_3_)_2_), 1.17 (d, *J*_H—H_= 9.0 Hz, 2.4H, CH(CH_3_)_2_), 1.35 (s, 2.4H, CH_3_), 1.79 (m, 1.2H, CH(CH_3_)_2_), 2.06 (s, 3.6H, CH_3_), 2.83 (m, 0.8, CH(CH_3_)_2_), 6.65–7.01 (m, 8H), 7.38 (m, 1H), 7.52 (m, 1H), 7.66 (m, 1H), 7.93 (m, 2H), 8.43 (m, 1H). ^13^C NMR (75 MHz, CDCl_3_, 298 K) δ (p.p.m.): 17.42, 18.62, 22.75, 22.97, 23.63, 24.25, 27.55, 28.90, 122.66, 122.77, 123.16, 123.26, 123.54, 123.60, 124.31, 124.73, 125.17, 127.20, 127.33, 127.46, 127.57, 127.86, 128.01, 128.91, 129.18, 131.35, 131.45, 131.92, 132.06, 133.64, 134.20, 134.60, 135.35, 135.50, 135.73, 145.40, 149.13, 156.77, 158.01, 159.37, 159.94 p.p.m..

### Refinement

The H atoms were positioned geometrically with C—H = 0.95 (aromatic carbon), 0.99 (methylene) and 0.98 (methyl) Å, and allowed to ride on their parent atoms with *U*_iso_(H) = 1.2 (1.5 for methyl) *U*_eq_(C).

### Crystal data

**Table:**

C_34_H_34_N_2_	*F*(000) = 1008
*M**_r_* = 470.63	*D*_x_ = 1.162 Mg m^−^^3^
Monoclinic, *P*2_1_/*c*	Mo *K*α radiation, λ = 0.71073 Å
Hall symbol: -P 2ybc	Cell parameters from 3054 reflections
*a* = 9.5495 (7) Å	θ = 2.4–26.0°
*b* = 16.4294 (12) Å	µ = 0.07 mm^−^^1^
*c* = 17.7237 (13) Å	*T* = 185 K
β = 104.579 (1)°	Block, red
*V* = 2691.2 (3) Å^3^	0.23 × 0.20 × 0.15 mm
*Z* = 4	

### Data collection

**Table:**

Bruker SMART CCD area-detector diffractometer	5304 independent reflections
Radiation source: fine-focus sealed tube	4587 reflections with *I* > 2σ(*I*)
Graphite monochromator	*R*_int_ = 0.038
phi and ω scan	θ_max_ = 26.1°, θ_min_ = 1.7°
Absorption correction: multi-scan (*ABSCOR*; Higashi, 1995)	*h* = −6→11
*T*_min_ = 0.985, *T*_max_ = 0.990	*k* = −20→20
14490 measured reflections	*l* = −21→21

### Refinement

**Table:**

Refinement on *F*^2^	Primary atom site location: structure-invariant direct methods
Least-squares matrix: full	Secondary atom site location: difference Fourier map
*R*[*F*^2^ > 2σ(*F*^2^)] = 0.083	Hydrogen site location: inferred from neighbouring sites
*wR*(*F*^2^) = 0.161	H-atom parameters constrained
*S* = 1.27	*w* = 1/[σ^2^(*F*_o_^2^) + (0.0304*P*)^2^ + 2.1478*P*] where *P* = (*F*_o_^2^ + 2*F*_c_^2^)/3
5304 reflections	(Δ/σ)_max_ < 0.001
331 parameters	Δρ_max_ = 0.21 e Å^−^^3^
0 restraints	Δρ_min_ = −0.24 e Å^−^^3^

### Special details

**Table:**

Geometry. All e.s.d.'s (except the e.s.d. in the dihedral angle between two l.s. planes) are estimated using the full covariance matrix. The cell e.s.d.'s are taken into account individually in the estimation of e.s.d.'s in distances, angles and torsion angles; correlations between e.s.d.'s in cell parameters are only used when they are defined by crystal symmetry. An approximate (isotropic) treatment of cell e.s.d.'s is used for estimating e.s.d.'s involving l.s. planes.
Refinement. Refinement of *F*^2^ against ALL reflections. The weighted *R*-factor *wR* and goodness of fit *S* are based on *F*^2^, conventional *R*-factors *R* are based on *F*, with *F* set to zero for negative *F*^2^. The threshold expression of *F*^2^ > σ(*F*^2^) is used only for calculating *R*-factors(gt) *etc*. and is not relevant to the choice of reflections for refinement. *R*-factors based on *F*^2^ are statistically about twice as large as those based on *F*, and *R*- factors based on ALL data will be even larger.

### Fractional atomic coordinates and isotropic or equivalent isotropic displacement parameters (Å^2^)

**Table:**

	*x*	*y*	*z*	*U*_iso_*/*U*_eq_	
N1	0.3180 (2)	0.67770 (13)	0.09099 (13)	0.0284 (5)	
N2	0.5846 (2)	0.75349 (12)	0.18313 (12)	0.0242 (5)	
C1	0.4448 (3)	0.64761 (15)	0.11007 (14)	0.0243 (5)	
C2	0.4616 (3)	0.55816 (15)	0.11074 (14)	0.0252 (5)	
C3	0.3482 (3)	0.50826 (16)	0.11938 (15)	0.0292 (6)	
H3	0.2607	0.5315	0.1219	0.035*	
C4	0.3650 (3)	0.42518 (17)	0.12425 (16)	0.0346 (7)	
H4	0.2889	0.3923	0.1299	0.042*	
C5	0.4951 (4)	0.39055 (17)	0.12071 (17)	0.0392 (7)	
H5	0.5070	0.3344	0.1245	0.047*	
C6	0.6079 (3)	0.43939 (17)	0.11159 (17)	0.0370 (7)	
H6	0.6950	0.4155	0.1093	0.044*	
C7	0.5933 (3)	0.52341 (16)	0.10582 (14)	0.0276 (6)	
C8	0.7101 (3)	0.57685 (16)	0.09295 (14)	0.0268 (6)	
C9	0.8246 (3)	0.54597 (18)	0.06549 (16)	0.0338 (6)	
H9	0.8280	0.4906	0.0554	0.041*	
C10	0.9324 (3)	0.59620 (19)	0.05315 (17)	0.0374 (7)	
H10	1.0081	0.5743	0.0354	0.045*	
C11	0.9286 (3)	0.6783 (2)	0.06693 (16)	0.0374 (7)	
H11	1.0005	0.7121	0.0576	0.045*	
C12	0.8174 (3)	0.71061 (18)	0.09474 (15)	0.0314 (6)	
H12	0.8160	0.7661	0.1048	0.038*	
C13	0.7073 (3)	0.66092 (16)	0.10781 (14)	0.0258 (6)	
C14	0.5844 (3)	0.69431 (15)	0.13656 (14)	0.0232 (5)	
C15	0.2838 (3)	0.76208 (16)	0.09064 (16)	0.0279 (6)	
C16	0.2400 (3)	0.79411 (18)	0.15418 (17)	0.0359 (7)	
C17	0.1844 (4)	0.8721 (2)	0.1478 (2)	0.0496 (9)	
H17	0.1548	0.8944	0.1895	0.060*	
C18	0.1717 (4)	0.9179 (2)	0.0808 (2)	0.0542 (9)	
H18	0.1334	0.9702	0.0776	0.065*	
C19	0.2160 (4)	0.88567 (18)	0.01948 (19)	0.0444 (8)	
H19	0.2077	0.9167	−0.0253	0.053*	
C20	0.2733 (3)	0.80749 (17)	0.02270 (16)	0.0322 (6)	
C21	0.2522 (4)	0.7434 (2)	0.22597 (19)	0.0509 (9)	
H21A	0.2089	0.7719	0.2617	0.076*	
H21B	0.3524	0.7332	0.2504	0.076*	
H21C	0.2030	0.6925	0.2118	0.076*	
C22	0.3218 (4)	0.77238 (19)	−0.04485 (17)	0.0437 (8)	
H22A	0.2704	0.7227	−0.0614	0.066*	
H22B	0.4238	0.7615	−0.0291	0.066*	
H22C	0.3022	0.8106	−0.0872	0.066*	
C23	0.7117 (3)	0.79637 (16)	0.22119 (15)	0.0258 (6)	
C24	0.8176 (3)	0.75988 (17)	0.28037 (15)	0.0293 (6)	
C25	0.9359 (3)	0.8071 (2)	0.31794 (18)	0.0397 (7)	
H25	1.0074	0.7840	0.3577	0.048*	
C26	0.9493 (3)	0.88710 (19)	0.29748 (19)	0.0421 (8)	
H26	1.0308	0.9169	0.3220	0.051*	
C27	0.8415 (3)	0.92263 (18)	0.24066 (18)	0.0379 (7)	
H27	0.8509	0.9768	0.2274	0.045*	
C28	0.7196 (3)	0.87959 (17)	0.20282 (15)	0.0306 (6)	
C29	0.5983 (4)	0.91871 (17)	0.14141 (17)	0.0383 (7)	
H29	0.5096	0.8886	0.1413	0.046*	
C30	0.6225 (5)	0.9105 (2)	0.06026 (18)	0.0586 (10)	
H30A	0.7107	0.9376	0.0585	0.088*	
H30B	0.5429	0.9349	0.0229	0.088*	
H30C	0.6291	0.8540	0.0481	0.088*	
C31	0.5708 (5)	1.0078 (2)	0.1589 (2)	0.0602 (10)	
H31A	0.6528	1.0402	0.1556	0.090*	
H31B	0.5563	1.0120	0.2104	0.090*	
H31C	0.4862	1.0271	0.1216	0.090*	
C32	0.8049 (3)	0.67254 (17)	0.30739 (16)	0.0349 (7)	
H32	0.7180	0.6488	0.2728	0.042*	
C33	0.9338 (4)	0.6197 (2)	0.3016 (2)	0.0543 (9)	
H33A	0.9457	0.6221	0.2494	0.082*	
H33B	0.9167	0.5644	0.3143	0.082*	
H33C	1.0199	0.6395	0.3374	0.082*	
C34	0.7840 (5)	0.6712 (2)	0.3902 (2)	0.0620 (11)	
H34A	0.8684	0.6931	0.4258	0.093*	
H34B	0.7692	0.6161	0.4047	0.093*	
H34C	0.7011	0.7035	0.3921	0.093*	

### Atomic displacement parameters (Å^2^)

**Table:**

	*U*^11^	*U*^22^	*U*^33^	*U*^12^	*U*^13^	*U*^23^
N1	0.0223 (11)	0.0285 (12)	0.0348 (12)	−0.0014 (10)	0.0080 (10)	−0.0008 (10)
N2	0.0220 (11)	0.0227 (11)	0.0268 (11)	0.0005 (9)	0.0040 (9)	0.0001 (9)
C1	0.0245 (13)	0.0280 (13)	0.0216 (12)	−0.0009 (11)	0.0079 (11)	−0.0022 (10)
C2	0.0276 (14)	0.0259 (13)	0.0208 (12)	−0.0028 (11)	0.0037 (11)	−0.0034 (10)
C3	0.0276 (14)	0.0309 (15)	0.0286 (13)	−0.0015 (12)	0.0060 (12)	0.0030 (11)
C4	0.0404 (17)	0.0307 (15)	0.0335 (15)	−0.0092 (13)	0.0108 (13)	−0.0011 (12)
C5	0.0532 (19)	0.0202 (14)	0.0457 (17)	0.0000 (13)	0.0149 (16)	−0.0009 (12)
C6	0.0336 (16)	0.0306 (15)	0.0465 (17)	0.0071 (13)	0.0094 (14)	−0.0010 (13)
C7	0.0257 (13)	0.0328 (15)	0.0228 (12)	0.0030 (11)	0.0033 (11)	−0.0012 (11)
C8	0.0230 (13)	0.0343 (15)	0.0208 (12)	0.0017 (11)	0.0011 (11)	−0.0008 (11)
C9	0.0311 (15)	0.0378 (16)	0.0313 (14)	0.0078 (13)	0.0054 (12)	−0.0028 (12)
C10	0.0252 (14)	0.0514 (19)	0.0371 (16)	0.0052 (13)	0.0104 (13)	−0.0059 (14)
C11	0.0242 (14)	0.0549 (19)	0.0343 (15)	−0.0090 (14)	0.0095 (12)	−0.0029 (14)
C12	0.0270 (14)	0.0360 (15)	0.0306 (14)	−0.0057 (12)	0.0061 (12)	−0.0038 (12)
C13	0.0222 (13)	0.0337 (15)	0.0200 (12)	−0.0020 (11)	0.0025 (10)	−0.0010 (11)
C14	0.0236 (13)	0.0220 (12)	0.0234 (12)	0.0026 (10)	0.0049 (10)	0.0029 (10)
C15	0.0179 (12)	0.0255 (14)	0.0390 (15)	−0.0013 (10)	0.0048 (11)	−0.0042 (11)
C16	0.0280 (15)	0.0394 (17)	0.0421 (16)	−0.0012 (13)	0.0124 (13)	−0.0076 (13)
C17	0.048 (2)	0.047 (2)	0.059 (2)	0.0048 (16)	0.0225 (17)	−0.0158 (17)
C18	0.057 (2)	0.0331 (18)	0.071 (2)	0.0147 (16)	0.0117 (19)	−0.0088 (17)
C19	0.0490 (19)	0.0307 (16)	0.0497 (18)	0.0041 (14)	0.0054 (16)	0.0028 (14)
C20	0.0246 (14)	0.0329 (15)	0.0365 (15)	−0.0009 (12)	0.0030 (12)	−0.0052 (12)
C21	0.051 (2)	0.061 (2)	0.0476 (19)	0.0000 (17)	0.0258 (17)	−0.0034 (17)
C22	0.052 (2)	0.0426 (18)	0.0359 (16)	0.0019 (15)	0.0105 (15)	−0.0037 (13)
C23	0.0237 (13)	0.0288 (14)	0.0266 (13)	−0.0023 (11)	0.0097 (11)	−0.0050 (11)
C24	0.0242 (14)	0.0349 (15)	0.0288 (13)	0.0026 (12)	0.0068 (11)	−0.0052 (12)
C25	0.0236 (14)	0.0531 (19)	0.0388 (16)	0.0065 (14)	0.0015 (13)	−0.0102 (14)
C26	0.0279 (15)	0.0450 (18)	0.0525 (19)	−0.0115 (14)	0.0085 (14)	−0.0197 (15)
C27	0.0385 (17)	0.0306 (15)	0.0485 (17)	−0.0128 (13)	0.0184 (15)	−0.0076 (13)
C28	0.0333 (15)	0.0348 (15)	0.0255 (13)	−0.0027 (12)	0.0108 (12)	−0.0036 (11)
C29	0.0500 (19)	0.0285 (15)	0.0358 (15)	−0.0028 (14)	0.0098 (14)	0.0040 (12)
C30	0.085 (3)	0.051 (2)	0.0367 (17)	0.007 (2)	0.0093 (19)	0.0064 (15)
C31	0.081 (3)	0.045 (2)	0.050 (2)	0.0152 (19)	0.007 (2)	0.0005 (16)
C32	0.0347 (16)	0.0362 (16)	0.0306 (14)	0.0080 (13)	0.0024 (13)	0.0027 (12)
C33	0.058 (2)	0.047 (2)	0.058 (2)	0.0203 (18)	0.0152 (19)	0.0099 (17)
C34	0.089 (3)	0.052 (2)	0.054 (2)	0.007 (2)	0.033 (2)	0.0065 (17)

### Geometric parameters (Å, º)

**Table:**

N1—C1	1.272 (3)	C19—H19	0.9300
N1—C15	1.424 (3)	C20—C22	1.503 (4)
N2—C14	1.275 (3)	C21—H21A	0.9600
N2—C23	1.418 (3)	C21—H21B	0.9600
C1—C2	1.478 (3)	C21—H21C	0.9600
C1—C14	1.507 (3)	C22—H22A	0.9600
C2—C3	1.397 (4)	C22—H22B	0.9600
C2—C7	1.404 (4)	C22—H22C	0.9600
C3—C4	1.374 (4)	C23—C24	1.396 (4)
C3—H3	0.9300	C23—C28	1.412 (4)
C4—C5	1.383 (4)	C24—C25	1.393 (4)
C4—H4	0.9300	C24—C32	1.527 (4)
C5—C6	1.384 (4)	C25—C26	1.379 (4)
C5—H5	0.9300	C25—H25	0.9300
C6—C7	1.389 (4)	C26—C27	1.376 (4)
C6—H6	0.9300	C26—H26	0.9300
C7—C8	1.481 (4)	C27—C28	1.383 (4)
C8—C9	1.399 (4)	C27—H27	0.9300
C8—C13	1.408 (4)	C28—C29	1.518 (4)
C9—C10	1.380 (4)	C29—C30	1.519 (4)
C9—H9	0.9300	C29—C31	1.532 (4)
C10—C11	1.373 (4)	C29—H29	0.9800
C10—H10	0.9300	C30—H30A	0.9600
C11—C12	1.384 (4)	C30—H30B	0.9600
C11—H11	0.9300	C30—H30C	0.9600
C12—C13	1.396 (4)	C31—H31A	0.9600
C12—H12	0.9300	C31—H31B	0.9600
C13—C14	1.496 (3)	C31—H31C	0.9600
C15—C20	1.398 (4)	C32—C34	1.530 (4)
C15—C16	1.399 (4)	C32—C33	1.531 (4)
C16—C17	1.380 (4)	C32—H32	0.9800
C16—C21	1.501 (4)	C33—H33A	0.9600
C17—C18	1.386 (5)	C33—H33B	0.9600
C17—H17	0.9300	C33—H33C	0.9600
C18—C19	1.369 (5)	C34—H34A	0.9600
C18—H18	0.9300	C34—H34B	0.9600
C19—C20	1.391 (4)	C34—H34C	0.9600
			
C1—N1—C15	125.5 (2)	H21A—C21—H21B	109.5
C14—N2—C23	123.3 (2)	C16—C21—H21C	109.5
N1—C1—C2	118.8 (2)	H21A—C21—H21C	109.5
N1—C1—C14	126.4 (2)	H21B—C21—H21C	109.5
C2—C1—C14	114.7 (2)	C20—C22—H22A	109.5
C3—C2—C7	120.0 (2)	C20—C22—H22B	109.5
C3—C2—C1	119.9 (2)	H22A—C22—H22B	109.5
C7—C2—C1	120.1 (2)	C20—C22—H22C	109.5
C4—C3—C2	120.6 (3)	H22A—C22—H22C	109.5
C4—C3—H3	119.7	H22B—C22—H22C	109.5
C2—C3—H3	119.7	C24—C23—C28	121.2 (2)
C3—C4—C5	119.8 (3)	C24—C23—N2	121.0 (2)
C3—C4—H4	120.1	C28—C23—N2	117.4 (2)
C5—C4—H4	120.1	C25—C24—C23	117.8 (3)
C4—C5—C6	120.1 (3)	C25—C24—C32	119.5 (3)
C4—C5—H5	120.0	C23—C24—C32	122.6 (2)
C6—C5—H5	120.0	C26—C25—C24	121.5 (3)
C5—C6—C7	121.2 (3)	C26—C25—H25	119.2
C5—C6—H6	119.4	C24—C25—H25	119.2
C7—C6—H6	119.4	C27—C26—C25	119.7 (3)
C6—C7—C2	118.3 (3)	C27—C26—H26	120.2
C6—C7—C8	122.4 (2)	C25—C26—H26	120.2
C2—C7—C8	119.3 (2)	C26—C27—C28	121.4 (3)
C9—C8—C13	118.3 (2)	C26—C27—H27	119.3
C9—C8—C7	121.5 (2)	C28—C27—H27	119.3
C13—C8—C7	120.2 (2)	C27—C28—C23	118.1 (3)
C10—C9—C8	121.2 (3)	C27—C28—C29	121.9 (3)
C10—C9—H9	119.4	C23—C28—C29	120.0 (2)
C8—C9—H9	119.4	C28—C29—C30	111.8 (3)
C11—C10—C9	120.3 (3)	C28—C29—C31	113.4 (3)
C11—C10—H10	119.8	C30—C29—C31	110.5 (3)
C9—C10—H10	119.8	C28—C29—H29	106.9
C10—C11—C12	119.8 (3)	C30—C29—H29	106.9
C10—C11—H11	120.1	C31—C29—H29	106.9
C12—C11—H11	120.1	C29—C30—H30A	109.5
C11—C12—C13	120.8 (3)	C29—C30—H30B	109.5
C11—C12—H12	119.6	H30A—C30—H30B	109.5
C13—C12—H12	119.6	C29—C30—H30C	109.5
C12—C13—C8	119.5 (2)	H30A—C30—H30C	109.5
C12—C13—C14	122.0 (2)	H30B—C30—H30C	109.5
C8—C13—C14	118.5 (2)	C29—C31—H31A	109.5
N2—C14—C13	128.8 (2)	C29—C31—H31B	109.5
N2—C14—C1	116.6 (2)	H31A—C31—H31B	109.5
C13—C14—C1	114.5 (2)	C29—C31—H31C	109.5
C20—C15—C16	121.4 (3)	H31A—C31—H31C	109.5
C20—C15—N1	119.3 (2)	H31B—C31—H31C	109.5
C16—C15—N1	118.5 (2)	C24—C32—C34	110.8 (2)
C17—C16—C15	118.1 (3)	C24—C32—C33	112.5 (3)
C17—C16—C21	122.0 (3)	C34—C32—C33	111.0 (3)
C15—C16—C21	119.9 (3)	C24—C32—H32	107.5
C16—C17—C18	121.5 (3)	C34—C32—H32	107.5
C16—C17—H17	119.2	C33—C32—H32	107.5
C18—C17—H17	119.2	C32—C33—H33A	109.5
C19—C18—C17	119.5 (3)	C32—C33—H33B	109.5
C19—C18—H18	120.2	H33A—C33—H33B	109.5
C17—C18—H18	120.2	C32—C33—H33C	109.5
C18—C19—C20	121.4 (3)	H33A—C33—H33C	109.5
C18—C19—H19	119.3	H33B—C33—H33C	109.5
C20—C19—H19	119.3	C32—C34—H34A	109.5
C19—C20—C15	118.0 (3)	C32—C34—H34B	109.5
C19—C20—C22	121.2 (3)	H34A—C34—H34B	109.5
C15—C20—C22	120.7 (3)	C32—C34—H34C	109.5
C16—C21—H21A	109.5	H34A—C34—H34C	109.5
C16—C21—H21B	109.5	H34B—C34—H34C	109.5
			
C15—N1—C1—C2	177.2 (2)	C2—C1—C14—C13	40.5 (3)
C15—N1—C1—C14	0.4 (4)	C1—N1—C15—C20	90.7 (3)
N1—C1—C2—C3	−23.6 (4)	C1—N1—C15—C16	−99.0 (3)
C14—C1—C2—C3	153.6 (2)	C20—C15—C16—C17	0.6 (4)
N1—C1—C2—C7	158.9 (2)	N1—C15—C16—C17	−169.5 (3)
C14—C1—C2—C7	−23.8 (3)	C20—C15—C16—C21	179.6 (3)
C7—C2—C3—C4	0.9 (4)	N1—C15—C16—C21	9.6 (4)
C1—C2—C3—C4	−176.5 (2)	C15—C16—C17—C18	0.0 (5)
C2—C3—C4—C5	0.1 (4)	C21—C16—C17—C18	−179.0 (3)
C3—C4—C5—C6	−0.6 (4)	C16—C17—C18—C19	−0.4 (5)
C4—C5—C6—C7	0.0 (5)	C17—C18—C19—C20	0.2 (5)
C5—C6—C7—C2	1.0 (4)	C18—C19—C20—C15	0.3 (5)
C5—C6—C7—C8	−177.5 (3)	C18—C19—C20—C22	−180.0 (3)
C3—C2—C7—C6	−1.5 (4)	C16—C15—C20—C19	−0.8 (4)
C1—C2—C7—C6	176.0 (2)	N1—C15—C20—C19	169.2 (3)
C3—C2—C7—C8	177.1 (2)	C16—C15—C20—C22	179.6 (3)
C1—C2—C7—C8	−5.4 (4)	N1—C15—C20—C22	−10.5 (4)
C6—C7—C8—C9	17.3 (4)	C14—N2—C23—C24	−70.3 (3)
C2—C7—C8—C9	−161.3 (2)	C14—N2—C23—C28	116.2 (3)
C6—C7—C8—C13	−163.2 (3)	C28—C23—C24—C25	−3.5 (4)
C2—C7—C8—C13	18.3 (4)	N2—C23—C24—C25	−176.7 (2)
C13—C8—C9—C10	0.0 (4)	C28—C23—C24—C32	173.6 (2)
C7—C8—C9—C10	179.6 (3)	N2—C23—C24—C32	0.5 (4)
C8—C9—C10—C11	−0.7 (4)	C23—C24—C25—C26	−0.4 (4)
C9—C10—C11—C12	1.2 (4)	C32—C24—C25—C26	−177.6 (3)
C10—C11—C12—C13	−1.1 (4)	C24—C25—C26—C27	2.4 (4)
C11—C12—C13—C8	0.4 (4)	C25—C26—C27—C28	−0.5 (4)
C11—C12—C13—C14	−179.0 (2)	C26—C27—C28—C23	−3.2 (4)
C9—C8—C13—C12	0.1 (4)	C26—C27—C28—C29	178.5 (3)
C7—C8—C13—C12	−179.4 (2)	C24—C23—C28—C27	5.3 (4)
C9—C8—C13—C14	179.5 (2)	N2—C23—C28—C27	178.7 (2)
C7—C8—C13—C14	−0.1 (3)	C24—C23—C28—C29	−176.4 (2)
C23—N2—C14—C13	−4.4 (4)	N2—C23—C28—C29	−3.0 (4)
C23—N2—C14—C1	171.4 (2)	C27—C28—C29—C30	89.9 (3)
C12—C13—C14—N2	−33.8 (4)	C23—C28—C29—C30	−88.4 (3)
C8—C13—C14—N2	146.8 (3)	C27—C28—C29—C31	−35.9 (4)
C12—C13—C14—C1	150.3 (2)	C23—C28—C29—C31	145.9 (3)
C8—C13—C14—C1	−29.0 (3)	C25—C24—C32—C34	65.4 (4)
N1—C1—C14—N2	41.1 (4)	C23—C24—C32—C34	−111.7 (3)
C2—C1—C14—N2	−135.9 (2)	C25—C24—C32—C33	−59.4 (3)
N1—C1—C14—C13	−142.5 (3)	C23—C24—C32—C33	123.5 (3)
